# T2 FLAIR Hyperintensity Volume Is Associated With Cognitive Function and Quality of Life in Clinically Stable Patients With Lower Grade Gliomas

**DOI:** 10.3389/fneur.2021.769345

**Published:** 2022-01-28

**Authors:** Tracy L. Luks, Javier E. Villanueva-Meyer, Christina Weyer-Jamora, Karin Gehring, Angela Jakary, Shawn L. Hervey-Jumper, Steve E. Braunstein, Paige M. Bracci, Melissa S. Brie, Ellen M. Smith, Susan M. Chang, Jennie W. Taylor

**Affiliations:** ^1^Department of Radiology and Biomedical Imaging, University of California, San Francisco, San Francisco, CA, United States; ^2^Zuckerberg San Francisco General Hospital, San Francisco, CA, United States; ^3^Department of Neurological Surgery, University of California, San Francisco, San Francisco, CA, United States; ^4^Department of Neurosurgery, Elisabeth-TweeSteden Hospital, Tilburg, Netherlands; ^5^Department of Cognitive Neuropsychology, Tilburg University, Tilburg, Netherlands; ^6^Department of Radiation Oncology, University of California, San Francisco, San Francisco, CA, United States; ^7^Department of Epidemiology and Biostatistics, University of California, San Francisco, San Francisco, CA, United States

**Keywords:** glioma, neuroimaging, neuro-oncology, neuropsychology, cognition

## Abstract

Survival outcomes for patients with lower grade gliomas (LrGG) continue to improve. However, damage caused both by tumor growth and by the consequences of treatment often leads to significantly impaired cognitive function and quality of life (QoL). While neuropsychological testing is not routine, serial clinical MRIs are standard of care for patients with LrGG. Thus, having a greater understanding of MRI indicators of cognitive and QoL impairment risk could be beneficial to patients and clinicians. In this work we sought to test the hypothesis that in clinically stable LrGG patients, T2 FLAIR hyperintensity volumes at the time of cognitive assessment are associated with impairments of cognitive function and QoL and could be used to help identify patients for cognitive and QoL assessments and interventions. We performed anatomical MR imaging, cognitive testing and QoL assessments cross-sectionally in 30 clinically stable grade 2 and 3 glioma patients with subjective cognitive concerns who were 6 or more months post-treatment. Larger post-surgical T2 FLAIR volume at testing was significantly associated with lower cognitive performance, while pre-surgical tumor volume was not. Older patients had lower cognitive performance than younger patients, even after accounting for normal age-related declines in performance. Patients with Astrocytoma, IDH mutant LrGGs were more likely to show lower cognitive performance than patients with Oligodendroglioma, IDH mutant 1p19q co-deleted LrGGs. Previous treatment with combined radiation and chemotherapy was associated with poorer self-reported QoL, including self-reported cognitive function. This study demonstrates the importance of appreciating that LrGG patients may experience impairments in cognitive function and QoL over their disease course, including during periods of otherwise sustained clinical stability. Imaging factors can be helpful in identifying vulnerable patients who would benefit from cognitive assessment and rehabilitation.

## Introduction

Survival outcomes for patients with lower grade (WHO grade 2 and 3) gliomas (LrGG) continue to improve as diagnosis and treatment evolve, with current median survival of 5–15 years (1). However, for patients with LrGG, tissue damage caused by tumor growth and by the consequences of treatment often leads to significantly impaired cognitive function (2–7). These cognitive impairments frequently have a negative impact on patients' QoL (8).

Within the LrGG patient population, there is high variability in the prevalence of cognitive and QoL impairment, and individual differences in the specific cognitive domains that are affected (9–15), although impairments in executive function, memory, and attention are most prevalent (11). Thus, it is challenging for clinicians to anticipate the extent to which a patient's cognition and QoL will be impacted. The timing of these impairments over the disease course is also unclear. Some reports suggest that cognitive impairments at the time of diagnosis resolve after acute recovery from surgery (e.g. within 3 months) (16, 17). However, others argue that practice effects are not always considered in those reports of improvement (18), and that while some recovery does occur, many patients remain impaired in specific cognitive functions and QoL domains (12). Additional impairment may emerge over the period of clinical stability following treatment due to longer term effects of radiation therapy, chemotherapy, and subtle tumor growth (19–23). Furthermore, impairments that were present since diagnosis or surgery may only become apparent to patients as they complete treatment and begin to resume the trajectories of their “normal” lives, re-encountering challenges in work, home, or family life.

The heterogeneity in the prevalence, nature and timing of impairments in cognitive function and QoL in patients with LrGG makes it difficult for clinicians, patients and caregivers to predict, monitor for, and respond to these impairments. The inclusion of cognitive function and QoL in LrGG patient treatment planning is of increasing interest as neuropsychological rehabilitation options expand (24). While neuropsychological testing is not routine, serial follow-up clinical MRIs are standard of care for patients with LrGG, so MRI indicators of cognitive and QoL impairment risk could be beneficial to patients and clinicians. For example, larger pre-surgical tumor volumes at diagnosis have been associated with poorer pre-surgical cognitive function and QoL in glioma (7, 25, 26), but the association between these pre-surgical volumes and longer term cognitive outcomes is unclear (27). To our knowledge, the associations between imaging measurements, cognitive function, and QoL in stable LrGG patients have not been investigated together in one study. In this work we sought to test the hypothesis that in clinically stable LrGG patients, T2 FLAIR hyperintensity volumes at the time of cognitive assessment are associated with impairments of cognitive function and QoL, and could be used to help identify patients for cognitive and QoL assessments and interventions.

## Methods

### Patients

Study participants were histologically confirmed LrGG (grade 2 and 3 glioma) patients who met the following inclusion criteria: (1) clinically stable and off treatment (i.e. surgery, radiation, chemotherapy) for at least 6 months, (2) expressed subjective cognitive concerns in discussion with their referring neuro-oncologist, (3) over 18 years old, (4) have a Karnofsky performance status (KPS) ≥ 70, (5) be fluent in English, (6) off steroids, and (7) have cognitive and motor function sufficient to complete the cognitive and QoL assessments. The restriction of referral based on subjective cognitive concerns was implemented because, in our experience, patients with cognitive concerns are more interested in participating in research that requires lengthy cognitive testing. Therefore, this inclusion criteria aimed to optimize the time referring neuro-oncologists were requested to spend discussing this study with patients.

Clinical stability was determined by the referring physician, based on lack of significant current growth in T2 FLAIR hyperintensity or T1 contrast-enhancing lesion, or worsening focal neurological symptoms. There was no upward limit on the time between completed treatment and enrollment, or the number or types of previous treatments. All participants gave written informed consent, and ethical approval was granted by the UCSF Institutional Review Board, in compliance with the Helsinki Declaration.

### Cognitive and QoL Assessment

Patients completed a one-hour computerized battery of standardized cognitive tests and QoL assessments within 1 month of a standard of care neuro-oncology appointment and MR imaging. The cognitive assessment included the adult Cognitive Domain tasks of the NIH Toolbox (http://www.nihtoolbox.org/Pages/default.aspx), a package that is validated, age-normed (28), and used in previous assessments of cognitive function in glioma patients (29, 30). The following cognitive domains were tested: spatial and episodic memory, processing speed, attention and executive functions, inhibition and attention, language comprehension and vocabulary, working memory, and reading and speech (see Table 1 for test details).

**Table 1:** Cognitive and quality of life assessment.

**Test name**	**Domain tested**	**Composite score**
Pattern comparison processing speed	Processing speed	Fluid cognition
Flanker inhibitory control and attention	Inhibition and attention	
Dimensional change card sort	Attention and executive functions	
Picture sequence memory	Spatial and episodic memory	
List sorting working memory	Working memory	
Oral reading recognition	Reading and speech	Crystallized cognition
Picture vocabulary	Language vocabulary and comprehension	

The NIH Toolbox provides raw scores and age-corrected scores. Because glioma patients vary widely in age, we used the age-corrected scores to control for the effects of normal aging on cognitive function. In addition to the 7 individual test scores, the NIH Toolbox provides a crystallized cognition composite score (based on vocabulary and reading recognition); a fluid cognition composite score (based on processing speed, attention, and working memory); and a total cognition score.

Crystallized abilities depend on knowledge and skills acquired through culture and education, often in childhood and adolescence, and are assessed with tasks of language comprehension, speech, vocabulary, and reading (31). Fluid abilities involve dynamic, flexible problem-solving, and are assessed with tasks of spatial memory, working memory, processing speed, executive function and attention.

QoL was assessed with the Functional Assessment of Cancer Therapy: Brain Cancer (FACT-Br, http://www.facit.org/FACITOrg/Questionnaires). The FACT-Br includes sub-scores for physical wellbeing, social wellbeing, emotional wellbeing, and functional wellbeing (which make up the FACT-G general cancer sub-score), cognition (brain cancer sub-score), and FACT-Br total scores (32). FACT-G scores and sub-scores were compared to norms reported by Holzner et al. (32). Published norms are not available for the Brain Cancer sub-score.

### Imaging

**Figure 1:**
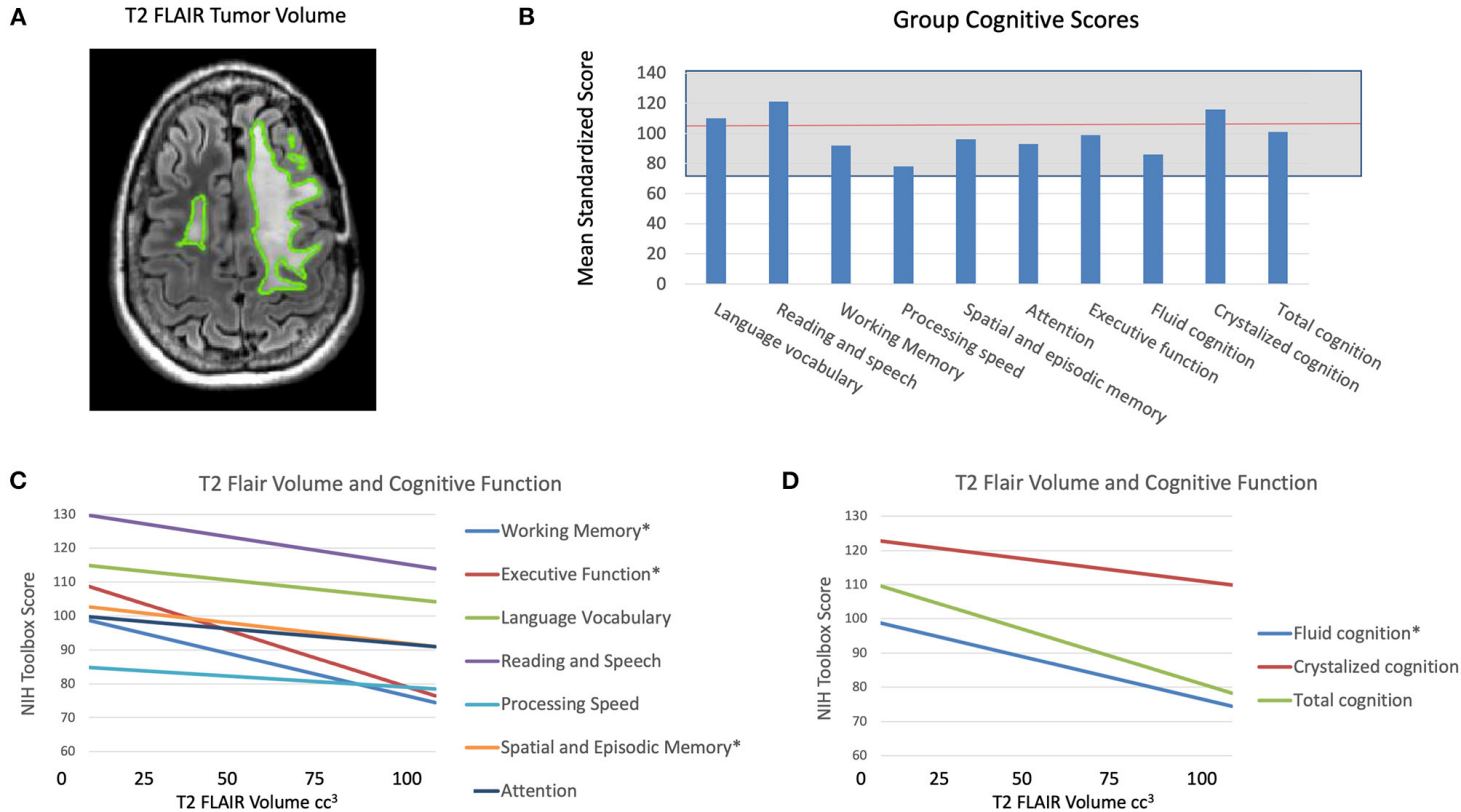
**(A)** Example of tumor volume defined by hyperintensity on T2 FLAIR MR images. **(B)** Mean age-corrected cognition scores from the NIH Toolbox, which has a healthy control mean of 100 and standard deviation of 10 (indicated by the gray box). **(C)** Mean age-corrected cognition subscores from the NIH Toolbox by T2 FLAIR tumor volume at the time of cognitive testing, *p < 0.05, corrected for multiple comparisons. **(D)** Mean age-corrected cognition summary scores from the NIH Toolbox by T2 FLAIR tumor volume at the time of cognitive testing, *p < 0.05, corrected for multiple comparisons.

Patients underwent standard of care MR imaging of the brain on a 3T MR scanner (GE Healthcare, Waukesha, WI, USA) with an 8- or 32-channel head coil within 1 month of the cognitive and QoL assessment (usually on the same day). The MR imaging protocol included standard of care T2-weighted Fluid Attenuated Inversion Recovery (FLAIR) and T1-weighted pre- and post-gadolinium sequences. Each patient's previous pre-surgical and post-surgical MRIs were also included for evaluation. Hyperintensity volumes were measured on T2 FLAIR images (only 1 patient had residual T1-weighted contrast enhancement). The T2 FLAIR hyperintensity volumes were defined using semi-automated software (3D Slicer 4; http://www.slicer.org) to include all the T2 FLAIR hyperintensity, relative to the surrounding normal tissue (Figure 1). Each volume was defined by a single investigator (TLL, AJ), with the guidance of a neuroradiologist (JEVM). The pre-surgical T2 FLAIR hyperintensity volume was used to determine the volume of tumor prior to the most recent surgery. The difference in volume between the pre- and post-surgical T2 FLAIR hyperintensity volumes was used to determine the Extent of Resection (EOR), and the change in T2 FLAIR hyperintensity volume from the post-surgical MRI to the time of cognitive and QoL assessment was used to measure tumor growth. Tumor location was categorized by hemisphere and primary lobe.

### Clinical and Demographic Variables

Clinical and demographic data were collected by review of patients' medical records including age, education, time since diagnosis, previous treatment, tumor grade, KPS and use of anti-epileptic drugs (AEDs). Patients were classified according to the 2021 WHO integrated diagnoses of lower-grade glioma subgroups: astrocytoma, *IDH* mutated; astrocytoma, *IDH* wildtype; oligodendroglioma, *IDH* mutated and 1p19q co-deleted; and NOS (molecular status unknown) (33). *IDH* mutation status and co-deletion of 1p19q were determined by immunohistochemistry and FISH, respectively (34).

### Statistical Analysis

The relationships between MR imaging characteristics, cognitive function and QoL scores were determined with ANOVAs and Spearman's rank correlations using JMP Pro 14 SAS software (http://www.jmp.com). Univariate analyses were performed for all variables. MR imaging characteristics included T2 FLAIR hyperintensity volume prior to surgery, T2 FLAIR hyperintensity volume at time of cognitive testing, EOR, T2 FLAIR hyperintensity growth, and tumor location (hemisphere and lobe). Clinical factors included age, WHO 2021 diagnosis, use of AEDs, prior chemotherapy, and prior combined radiation and chemotherapy. To correct for the 14 univariate analyses completed for all cognitive and QoL outcomes, we used the Benjamin-Hochberg False Discovery Rate (FDR = 0.2) threshold correction (35). Multivariate linear regression analyses were used to examine interactive effects of T2 FLAIR hyperintensity volume and clinical factors on cognition and QoL. These were exploratory analyses, given the small sample size, and no corrections were made for multiple comparisons.

## Results

### Patient Demographics and Clinical Factors

Thirty patients with histologically confirmed LrGG were enrolled. Complete cognitive function data were collected for 27 patients and complete QoL data were collected for 28, due to equipment errors (the missing cognitive and QoL data were from different patients). Eighteen patients were diagnosed as WHO grade 2, and 12 patients were diagnosed as WHO grade 3. Twelve were diagnosed as astrocytoma, *IDH* mutated*;* 14 as oligodendroglioma, *IDH* mutated and 1p19q codeleted; and four as NOS (owing to unknown IDH or 1p19q status). Median age at enrollment was 45 years old (range 26–66) and median time from diagnosis was 72 months (range 20–204). Twenty-two patients had 1 previous surgery, 3 patients had 2 previous surgeries, 3 patients had 3 previous surgeries, and 2 patients had only previous biopsies (due to tumor location). Seventeen patients had received combined radiation and chemotherapy, 7 chemotherapy alone, and 6 neither. Twenty-one patients were on AEDs at the time of testing. No patients reported seizures in the last 6 months. Additional clinical characteristics and demographics are reported in Table 2.

**Table 2:** Patient demographics and clinical factors.

Median age, years (range)	45 (26–66)
Median education, years (range)	16 (8–20)
Gender, *n* (%)	
Male	11 (37)
Female	19 (63)
Median time since diagnosis, months (range)	72 (20–204)
Median time since last surgery, months (range)	43 (8–139)
KPS, *n* (%)	
100	4 (13)
90	15 (50)
80	10 (33)
70	1 (3)
Prior Treatment, *n* (%)	
Surgery only	6 (20)
Surgery and chemotherapy	7 (23)
Surgery, chemotherapy and radiation therapy	17 (57)
Median time since last chemotherapy, months (range)	30 (6–98)
Median time since radiation therapy, months (range)	36 (6–139)
Anti-epileptic medication at testing, *n* (%)	
Yes	21 (70)
No	9 (30)
WHO 2021 diagnosis, *n* (%)	
Oligodendroglioma, *IDH* mutant, 1p19q co-deleted	14 (47)
Grade 2	10
Grade 3	4
Astrocytoma, *IDH* mutant	12 (40)
Grade 2	7
Grade 3	5
NOS	4 (13)
Grade 2	1
Grade 3	3

### Imaging Characteristics

At the time of testing, T2 FLAIR hyperintensity volume mean (range) was 26.7 cc^3^ (0.1–96). T2 FLAIR hyperintensity pre-surgery volume mean was 41.9 cc^3^ (1–147.9), volumetric EOR mean was 74% (0–100%, where 0 = biopsy), and mean T2 FLAIR hyperintensity volume growth from the post-surgical MRI to the time of the cognitive and QoL assessment was 15.1 cc^3^ (−35.7–68.7). Two patients had bilateral tumors, 17 patients had left hemisphere tumors, and 11 patients had right hemisphere tumors. Twenty were frontal, 7 were parietal, 2 were temporal, and 1 was occipital (Table 3).

**Table 3:** Imaging characteristics.

**Imaging measures**	
T2 FLAIR hyperintensity volume pre-surgery (mean, range)	41.9 cc^3^ (1–147.9)
Extent of resection (mean, range)	74% (0–100%)
T2 FLAIR hyperintensity volume at testing (mean, range)	26.7 cc^3^ (0.1–96)
T2 FLAIR hyperintensity volume growth (mean, range)	15.1 cc^3^ (−35.7–68.7)
**Tumor location**	
Bilateral (*n =* 2)	Frontal *n =* 2
Left hemisphere (*n =* 17)	Frontal *n =* 10Parietal *n =* 5Temporal *n =* 2
Right hemisphere (*n =* 11)	Frontal *n =* 8Parietal *n =* 2Occipital *n =* 1

Previous combined radiation and chemotherapy was significantly associated with larger T2 FLAIR hyperintensity volume at time of testing (F = 9.7, *p* = 0.004), and T2 FLAIR hyperintensity volume growth (F = 8.9, *p* = 0.006). Imaging variables were not significantly associated with any other demographic or clinical variables.

### Cognitive Functioning

For the patients as a group, Processing speed was ≥ −1 SD below average (all NIH Toolbox standardized age-corrected scores have a mean of 100 and standard deviation of 15), with mean standard score = 78 (range 40–111). Sixteen patients (53%) scored ≥1 SD below average on Processing speed. No other measures were ≥ −1 SD below average for the group as a whole. The group Fluid cognition mean standard score was 86 (range 58–113) and the group Crystallized cognition mean standard score was 116 (range 75–141). Thirteen patients (43%) scored ≥1 SD below average on Fluid cognition. Twenty patients (67%) scored ≥1 SD below average on at least one test (Table 4, Supplementary Table 1, Figure 1).

**Table 4:** Cognitive and quality of life scores.

**Cognitive domain and QoL scores**	**Mean score (range)**	**T2 FLAIR volume at testing**	**Pre-surgical T2 FLAIR volume**	**Extent of resection**	**T2 FLAIR volume growth**
Processing speed	78 (40–111)	F = 3.5, *p =* 0.073	F = 0.005,*p =* 0.99	F = 2.9,*p =* 0.1	F = 2.01,*p =* 0.16
Attention	93 (55–135)	F = 3.7, *p =* 0.066	F = 0.1,*p =* 0.76	F = 0.1,*p =* 0.75	F = 3.4,*p =* 0.078
Working memory	92 (−114)	F = 9.87, *p =* 0.004	F = 2.8,*p =* 0.11	F = 1.7,*p =* 0.2	F = 5.4,*p =* 0.029
Spatial and episodic memory	96 (72–146)	F = 3.26, *p =* 0.084	F = 1.27,*p =* 0.27	F = 2.7,*p =* 0.11	F = 0.77,*p =* 0.39
Executive function	99 (57–134)	F = 4.18, *p =* 0.05	F = 0.22,*p =* 0.65	F = 0.06,*p =* 0.8	F = 2.8,*p =* 0.11
Language vocabulary	110 (74–140)	F = 1.3, *p =* 0.256	F = 1.5,*p =* 0.23	F = 0.9,*p =* 0.35	F = 0.16,*p =* 0.69
Reading and speech	121 (76–150)	F = 5.14 *p =* 0.033	F = 1.7,*p =* 0.2	F = 3.5,*p =* 0.07	F = 1.6,*p =* 0.22
Fluid cognition	86 (58–113)	F = 8.6, *p =* 0.007	F = 0.22,*p =* 0.64	F = 1.2,*p =* 0.28	F = 4.8,*p =* 0.038
Crystallized cognition	116 (75–141)	F = 2.7, *p =* 0.11	F = 1.46,*p =* 0.24	F = 1.8,*p =* 0.19	F = 0.55,*p =* 0.47
Total cognition	101 (67–126)	F = 6.4, *p =* 0.019	F = 0.88,*p =* 0.36	F = 1.9,*p =* 0.18	F = 2.4,*p =* 0.13
FACT functional wellbeing	13 (7–23)	F = 0.26, *p =* 0.62	F = 0.31,*p =* 0.58	F = 0.16,*p =* 0.69	F = 0.18,*p =* 0.68
FACT physical wellbeing	23 (14–28)	F = 0.06, *p =* 0.8	F = 0.5,*p =* 0.49	F = 0.01,*p =* 0.98	F = 0.27,*p =* 0.61
FACT emotional wellbeing	18 (7–25)	F = 0.7, *p =* 0.41	F = 0.04,*p =* 0.84	F = 0.01,*p =* 0.99	F = 0.72,*p =* 0.40
FACT social wellbeing	20 (2–28)	F = 0.05, *p =* 0.82	F = 0.8,*p =* 0.38	F = 0.04,*p =* 0.84	F = 0.24,*p =* 0.63
FACT brain cancer (cognition)	63 (43–77)	F = 1.23, *p =* 0.28	F = 0.52,*p =* 0.48	F = 0.35,*p =* 0.56	F = 3.3,*p =* 0.08
Fact G (general)	74 (57–91)	F = 0.04, *p =* 0.85	F = 0.17,*p =* 0.68	F = 0.06,*p =* 0.8	F = 0.13,*p =* 0.72
Fact Br total	137 (100–165)	F = 0.26, *p =* 0.62	F = 0.39,*p =* 0.54	F = 0.04,*p =* 0.85	F = 0.59,*p =* 0.45

*NIH toolbox: Standardized Age-corrected scores, where 100 = average, and 1 standard deviation = 15 points. Higher FACT-Br scores indicated greater self-reported wellbeing. T2 FLAIR Volume Growth = the change in T2 FLAIR hyperintensity volume from the post-surgical MRI to the time of cognitive and QoL assessment*.

### Larger T2 FLAIR Hyperintensity Volume Is Associated With Lower Cognitive Functioning

Larger T2 FLAIR hyperintensity volume at time of testing was significantly negatively associated with age-corrected performance on several tests, including: (1) List Sorting Working Memory Test (working memory) (F = 9.87, *p* = 0.004); (2) Dimensional Change Card Sort Test (attention and executive functions) (F = 4.67, *p* = 0.05); (3) Fluid cognition sub-scores (F = 8.6, *p* = 0.007); and (4) Total cognition score (F = 6.4, *p* = 0.019) (Figures 1B,C). There were no significant associations between cognitive function and most recent pre-surgical T2 FLAIR hyperintensity volumes or EOR. Larger T2 FLAIR hyperintensity growth was significantly associated with lower age-corrected performance on the List Sorting Working Memory Test (F = 5.4, *p* = 0.029) and Fluid cognition sub-scores (F = 4.8, *p* = 0.038). There were no significant associations between tumor location (hemisphere or lobe) and cognitive function. When controlling for treatment history, there remain significant effects of T2 FLAIR hyperintensity volume on the List Sorting Working Memory Test (working memory) (F = 8.95, *p* = 0.007), Fluid cognition sub-scores (F = 6.19, *p* = 0.02), and Total cognition score (F = 5.85, *p* = 0.025). There was significant interactive effect between treatment history and T2 FLAIR hyperintensity volume on the List Sorting Working Memory Test (F = 5.63, *p* = 0.011). The association of larger T2 FLAIR volume in patients with worse working memory was in fact strongest in patients with no previous chemotherapy or radiation therapy. There were no other significant statistical interactions between the effects of imaging variables and clinical variables on cognitive function.

### Increasing Age and Molecular Subgroup Associated With Lower Cognitive Functioning

**Figure 2:**
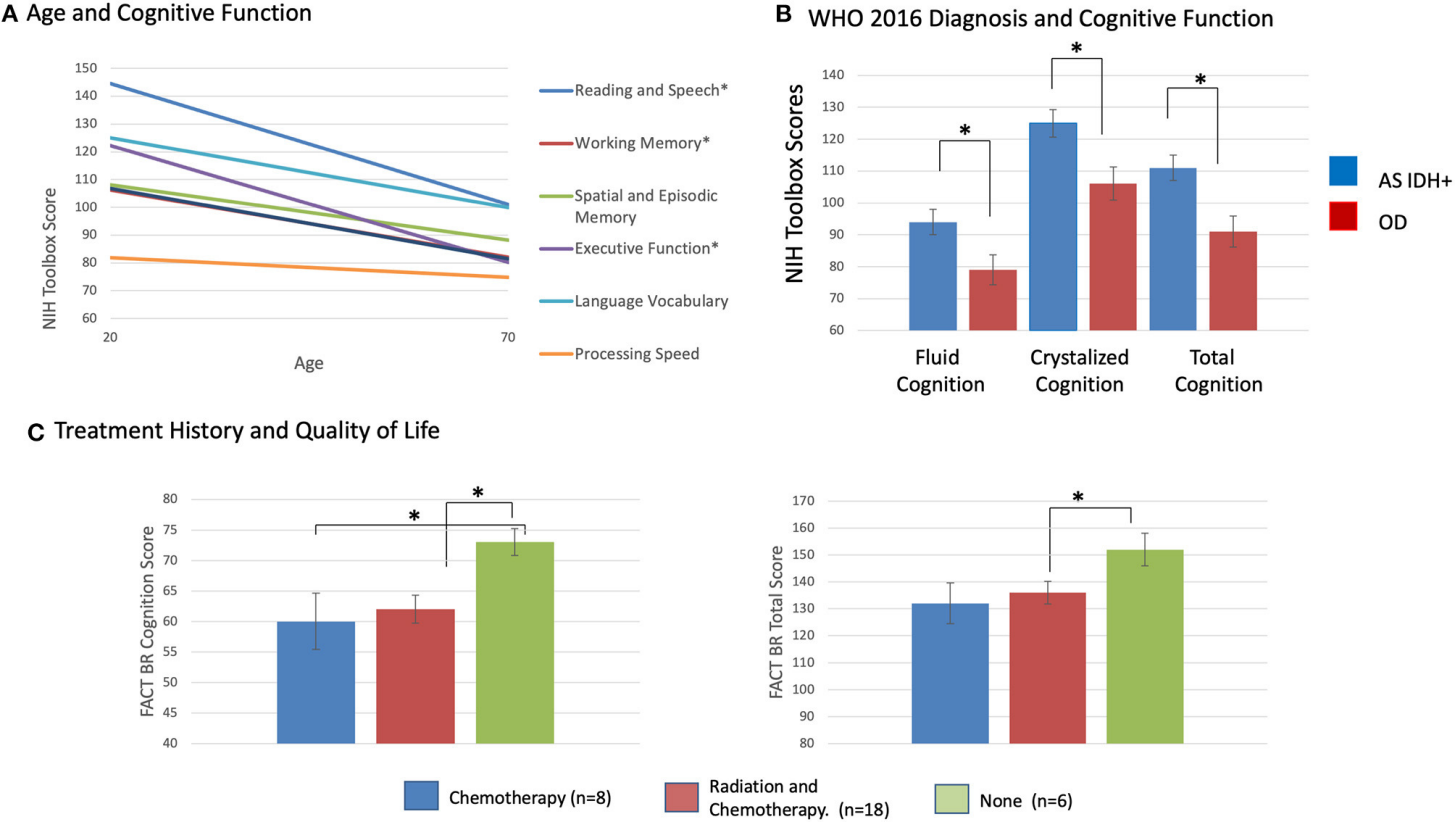
**(A)** Mean age-corrected cognition subscores from the NIH Toolbox by patient age, *p < 0.05, corrected for multiple comparisons. **(B)** Mean age-corrected cognition summary scores from the NIH Toolbox by WHO 2021 molecular diagnostic subgroup, *p 0.05, corrected for multiple comparisons. **(C)** Mean FACT-BR cognition subscore, FACT-BR Total score by patient radiation and chemotherapy history, *p < 0.05, corrected for multiple comparisons.

Increasing age at testing significantly correlated with lower age-corrected performance on several tests, including: (1) Oral Reading Recognition Test (reading and speech) (F = 10.7, *p* = 0.003); (2) List Sorting Working Memory Test (working memory) (F = 8.0, *p* = 0.009); (3) Dimensional Change Card Sort Test (attention and executive functions) (F = 8.19, *p* = 0.009); (4) Fluid cognition sub-score (F = 4.5, *p* = 0.045); (5) Crystallized cognition sub-score (F = 6.3, *p* = 0.02); and (6) Total cognition score (F = 7.22, *p* = 0.013). Older patients performed lower than younger patients in all these domains, even after accounting for normal age-related declines in performance (Figure 2).

Patients with astrocytoma, *IDH* mutated LrGGs had significantly worse age-corrected performance than patients with oligodendrogliomas, *IDH* mutated 1p19q co-deleted LrGGs on: (1) Oral Reading Recognition Test (reading and speech) (F = 5.02, *p* = 0.036); (2) Fluid cognition scores (F = 5.44, *p* = 0.03); 3) Crystallized cognition scores (F = 7.67, *p* = 0.012), and (4) Total cognition scores (F = 9.63, *p* = 0.006) (Figure 2). There were no significant statistical interactions between the effects of WHO 2021 diagnosis and grade on cognitive scores. There were no significant relationships between cognitive performance, time from diagnosis, grade, AED use, or previous treatment history.

### Previous Treatment Associated With Lower QoL Scores

Eleven patients (37%) scored worse than 1 SD below normal on the FACT-G total. Twenty-eight patients (93%) scored lower than 1 SD below normal on at least one subscale (all but 2 patients), including 25 patients on the functional wellbeing subscale (Table 4, Supplementary Table 2). There were no significant associations between T2 FLAIR hyperintensity volumes at testing, pre-surgical T2 FLAIR hyperintensity volumes, EOR, T2 FLAIR hyperintensity growth or lobe, and FACT-Br scores. There were no significant statistical interactions between the effects of imaging variables and clinical variables on FACT-Br scores.

Previous treatment with chemotherapy was associated with lower self-reported cognitive function scores (F = 6.5, *p* = 0.017) and lower FACT-Br total scores (F = 4.3, *p* = 0.049) (Figure 2). Previous treatment with combined radiation and chemotherapy was associated with lower self-reported cognition scores (F = 6.1, *p* = 0.02) and social wellbeing scores (F = 5.5, *p* = 0.027). There were no significant relationships between QoL scores and age, grade, AED use, time since diagnosis, WHO 2021 subgroup diagnosis. There were no significant correlations between FACT-Br scores and NIH Toolbox Cognitive scores. This includes a lack of relationship between the FACT-BrC (self-reported cognition) and the objective NIH Toolbox cognitive measures, and between those measures and the FACT-BR emotional wellbeing scores (which includes questions about mood) and FACT-BR physical wellbeing scores (which includes questions about fatigue). However, there were significant correlations between between self-reported cognition (FACT-BrC) and physical wellbeing (Spearmans' rho = 0.64, *p* = 0.003) and between FACT-BrC and FACT-G scores (Spearmans' rho = 0.68, *p* = 0.001).

## Discussion

In this study, we evaluated objective cognitive test performance and QoL in 30 clinically stable LrGG patients who reported subjective cognitive concerns. The aim of this research was to identify imaging factors in clinically stable LrGG patients associated with cognitive and QoL impairments. Given that most patients demonstrated significant impairment in at least one cognitive and/or one QoL domain at a median of 6 years after diagnosis, this study highlights the continued importance of work in this area.

We identified imaging factors associated with lower cognitive function and QoL that may be useful when monitoring patients with these concerns. Larger T2 FLAIR hyperintensity volume at testing was associated with lower cognitive functioning in the domains of working memory, executive functioning, and processing speed, while performance on these domains did not correlate with pre-surgical tumor volume, or EOR. These associations were not explained by clinical factors such as treatment history, grade or molecular subgroup. The association with working memory was in fact strongest in patients with no previous chemotherapy or radiation therapy, indicating that it was not simply a by-product of the effects of radiation therapy, which is more likely to be used in patients with larger tumors. These significant associations are of particular interest as they are noted even on tests for which the group as a whole performed normally, indicating that individual differences in T2 FLAIR hyperintensity volumes vary with cognitive performance. Even in clinically stable patients, larger tumor volume may cause more disruption of functional networks, and alterations in structural and functional connectivity have been associated with cognitive function in glioma (36–41).

Older patients were more likely to have lower cognitive performance, even after accounting for normal age-related declines in performance, indicating that the cognitive impact of glioma increases with age. Patients with astrocytoma, *IDH* mutated LrGGs were more likely to have lower executive function scores and FACT-Br cognition scores than patients with oligodendroglioma, *IDH* mutated 1p19q-codeleted. Wefel et al. (42) found that *IDH* wild-type pre-surgical grade 3 and 4 glioma patients had lower cognitive scores than IDH mutated patients, and hypothesized this difference may be related to the more aggressive proliferation and dispersion characteristics of *IDH* wild-type tumors. Similar results have been reported by Derks et al. (43) and van Kessel et al. (25). Zhang et al. (44) incorporated 1p19q and IDH mutation status to classify pre-surgical LrGG patients as astrocytoma, *IDH* mutated or oligodendroglioma, *IDH* mutated 1p19q-codeleted and found, similar to the present study, that working memory scores were higher in the astrocytoma group than the oligodendroglioma group.

We also found that treatment with combined radiation and chemotherapy (compared to surgery only) was associated with worse self-reported QoL, including self-reported cognitive function. These results are consistent with reports that radiation therapy and chemotherapy can cause progressive cognitive decline (23, 45). Future studies with larger sample sizes will be necessary to determine the independent associations between cognitive function, molecular status and treatment history. However, even if the sample was large enough to include several potential covariates, it remains difficult to identify the separate contributions of these factors, because many treatment decisions are clinically driven by molecular status and grade.

It is also interesting to note which factors were not significantly associated with cognitive performance and QoL. Tumor laterality, for example, was not significantly associated with cognitive task performance, even for language tests. This suggests that these slow-growing gliomas impact broad functional networks, and that cognition may be preserved by compensatory re-organization during that period of slow growth. Although previous treatment with combined radiation and chemotherapy was associated with worse self-reported cognitive function, treatment was not associated with lower performance on cognitive tasks. Furthermore, there were no significant correlations between scores on cognitive tasks and self-reported QoL cognitive scores, indicating the complementary value of both types of assessment. The FACT-Br and other self-report measures ask patients to limit their responses to their experience in a recent time window (e.g. last 7 days), which may not be reflective of their broader QoL experience. However, low associations between cognitive performance and self-perceived cognitive deficits have been reported across oncological populations, where self-perceived cognitive symptoms are more closely related to self-reported symptoms of physical and psychological distress, such as fatigue and depression (46–49). Similarly, in our study, there were significant correlations between self-reported cognitive symptoms on the FACT-BrC and the physical wellbeing score and FACT-G score (summary of physical, emotional, social and functional wellbeing).

As mentioned above, sample size limits this study from examining the full effects of some factors, such as tumor location within hemisphere, and limits power to detect the main and interacting effects of the variables that were examined, such as WHO 2021 diagnosis, grade and treatment. Our patient sample is also biased both by the use of subjective cognitive concerns for study referral, and by the exclusion of patients with objective cognitive impairments so severe they cannot complete the assessments, and therefore our results may not reflect the associations between imaging factors, cognition and QoL in the total population of LrGG. The inclusion of cognition and QoL outcomes in future studies of clinically stable patients and in clinical trials of new therapeutic agents and treatment protocols will allow larger more representative patient groups, and longitudinal multifactorial analyses.

Given the emerging research for the use of cognitive rehabilitation for addressing cognitive and QoL concerns in LrGG (50–53), this study underscores the value of including cognitive and QoL assessments as part of LrGG care, as well as highlighting the groundwork needed to refine cognitive treatments. More specifically, while still in its infancy for use in LrGG, cognitive rehabilitation models may want to consider how clinical factors such as age, T2 FLAIR volumes, tumor molecular characteristics, and treatment effects may jointly influence treatment response.

In conclusion, it is important to appreciate that as LrGG becomes a disease with sustained periods of clinical stability, many of these patients are exhibiting significant impairments in cognitive function and QoL. We have found that larger current T2 FLAIR hyperintensity volume, older age, a diagnosis of astrocytoma, and adjuvant treatment are associated with lower cognitive functioning and QoL, and such clinical factors could help to identify LrGG patients in need of cognitive assessment and intervention.

## Data Availability Statement

The datasets presented in this article are not readily available because Data contain PHI. Requests to access the datasets should be directed to Tracy.Luks@ucsf.edu.

## Ethics Statement

The studies involving human participants were reviewed and approved by University of California San Francisco IRB. The patients/participants provided their written informed consent to participate in this study.

## Author Contributions

TL, SC, KG, and JT designed the study. TL, JV-M, AJ, and JT carried out the data acquisition. TL analyzed the data. All authors participated in the interpretation of the results and the manuscript writing.

## Funding

This research was funded by the American Cancer Society (to JT and TL); the LoGlio Collective (to all authors); the Sheri Sobrato Brisson Brain Cancer Fund (to CW-J, SC, and SH-J); Robert Wood Johnson Foundation [74259] (to SH-J); and National Institutes of Health [NINDS K08 110919-01] (to SH-J).

## Conflict of Interest

The authors declare that the research was conducted in the absence of any commercial or financial relationships that could be construed as a potential conflict of interest.

## Publisher's Note

All claims expressed in this article are solely those of the authors and do not necessarily represent those of their affiliated organizations, or those of the publisher, the editors and the reviewers. Any product that may be evaluated in this article, or claim that may be made by its manufacturer, is not guaranteed or endorsed by the publisher.

## Supplementary Material

Supplemental Figure 1 Age-corrected cognition subscores from the NIH Toolbox by patient age.

Supplementary Table 1 Cognitive functioning.

Supplementary Table 2 Quality of life.
