# Supplementary figures and images for: T2 FLAIR Hyperintensity Volume Is Associated With Cognitive Function and Quality of Life in Clinically Stable Patients With Lower Grade Gliomas
Source: Front Neurol. 2022 Jan 28;12:769345. doi: 10.3389/fneur.2021.769345 (PMC8831734; doi:10.3389/fneur.2021.769345)

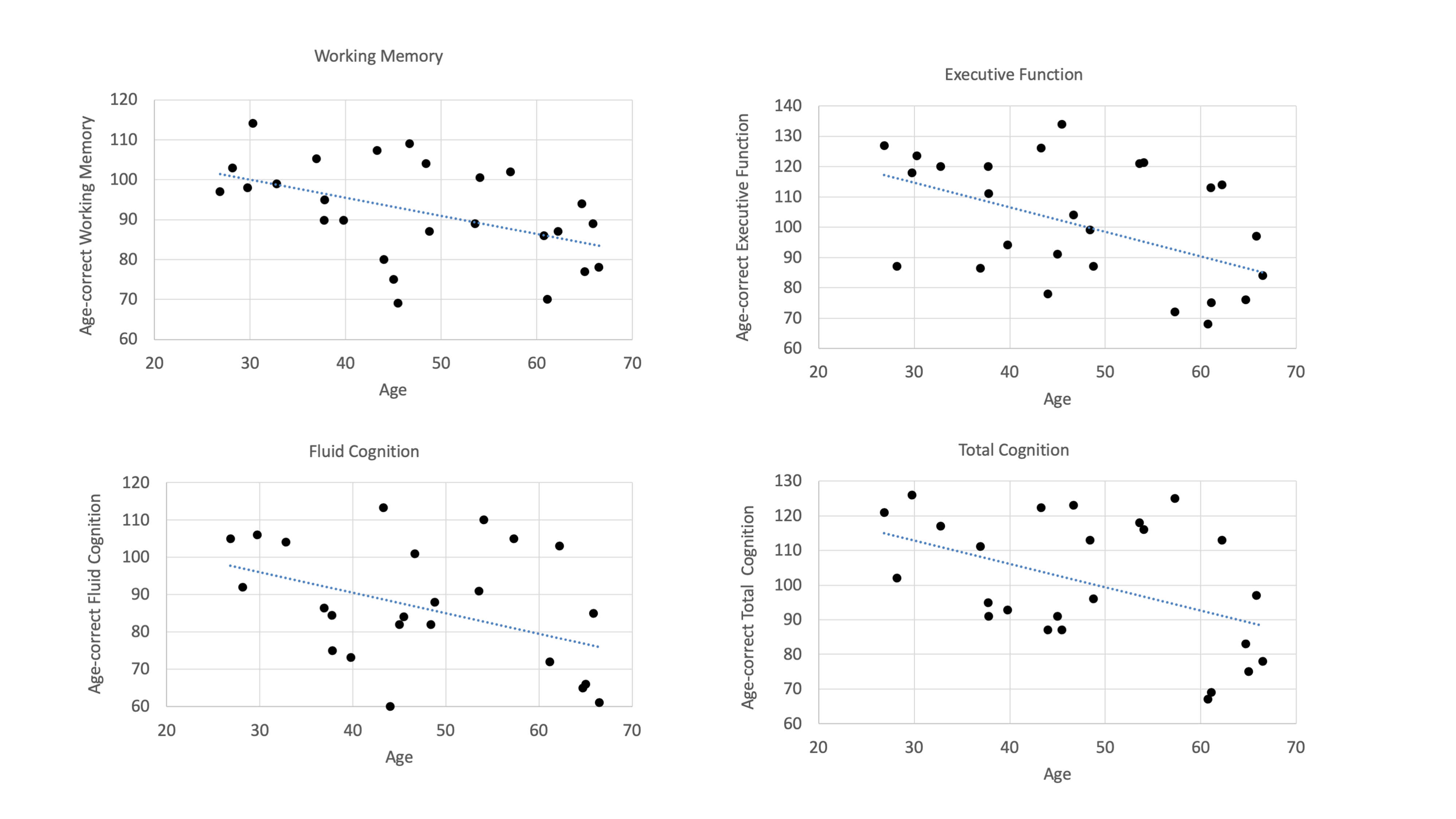

Supplement: Supplemental Figure 1 — Age-corrected cognition subscores from the NIH Toolbox by patient age. [file Image_1.TIF]
