# Supplementary material for: T2 FLAIR Hyperintensity Volume Is Associated With Cognitive Function and Quality of Life in Clinically Stable Patients With Lower Grade Gliomas
Source: Front Neurol. 2022 Jan 28;12:769345. doi: 10.3389/fneur.2021.769345 (PMC8831734; doi:10.3389/fneur.2021.769345)
Supplement: Supplementary Table 1 — Cognitive functioning. [file Table_1.docx]

Supplementary Table 1.

| **NIH Toolbox Domain** | **Mean** (range) | **Number of patients** | | |
| --- | --- | --- | --- | --- |
|  |  | **< 1 SD** (%) | **< 1.5 SD** (%) | **< 2 SD** (%) |

| Processing speed | 78(40-111) | 16 (53%) | 8 (27%) | 8 (27%) |
| --- | --- | --- | --- | --- |
| Attention | 93 (55-135) | 11 (37%) | 4 (13%) | 3 (10%) |
| Working memory | 92 (-114) | 5 (17%) | 4 (13%) | 0 (0%) |
| Spatial and episodic memory | 96 (72-146) | 8 (27%) | 4 (13%) | 0 (0%) |
| Executive function | 99 (57-134) | 7 (23%) | 6 (20%) | 2 (7%) |
| Language vocabulary | 110 (74-140) | 3 (10%) | 2 (7%) | 0 (0%) |
| Reading and speech | 121 (76-150) | 2 (7%) | 1 (3%) | 0 (0%) |
| Fluid cognition | 86 (58-113) | 13 (43%) | 7 (23%) | 5 (17%) |
| Crystallized cognition | 116 (75-141) | 2 (7%) | 1 (3%) | 0 (0%) |
| Total cognition | 101 (67-126) | 4 (13%) | 3 (10%) | 2 (7%) |

NIH toolbox: Standardized Age-corrected scores, where 100 = average, and 1 standard deviation = 15 points.

Supplementary Table 2.

| **FACT-Br** | **Mean** (range) | **Number of patients** | | |
| --- | --- | --- | --- | --- |
|  |  | **< 1 SD** (%) | **< 1.5 SD** (%) | **< 2 SD** (%) |
| **Functional well-being** | 13 (7-23) | 25 (83%) | 13 (43%) | 3 (10%) |
| **Fact G (general)** | 23 (14-28) | 11 (37%) | 6 (20%) | 0 |
| **Physical well-being** | 18 (7-25) | 7 (23%) | 2 (7%) | 1 (3%) |
| **Emotional well-being** | 20 (2-28) | 5 (17%) | 5 (17%) | 2 (7%) |
| **Social well-being** | 63 (43-77) | 3 (10%) | 2 (7%) | 0 |
| **Brain Cancer (cognition)** | 74 (57-91) | na | na | na |
| **Fact BR total** | 137 (100-165) | na | na | na |
